# Effect of Infant RSV Infection on Memory T Cell Responses at Age 2-3 Years

**DOI:** 10.3389/fimmu.2022.826666

**Published:** 2022-03-17

**Authors:** Tatiana Chirkova, Christian Rosas-Salazar, Tebeb Gebretsadik, Samadhan J. Jadhao, James D. Chappell, R. Stokes Peebles, William D. Dupont, Dawn C. Newcomb, Sergejs Berdnikovs, Peter J. Gergen, Tina V. Hartert, Larry J. Anderson

**Affiliations:** ^1^ Department of Pediatrics, Emory University School of Medicine and Children’s Healthcare of Atlanta, Atlanta, GA, United States; ^2^ Department of Pediatrics, Vanderbilt University Medical Center, Nashville, TN, United States; ^3^ Department of Biostatistics, Vanderbilt University Medical Center, Nashville, TN, United States; ^4^ Department of Medicine, Vanderbilt University Medical Center, Nashville, TN, United States; ^5^ Department of Medicine, Northwestern University Feinberg School of Medicine, Chicago, IL, United States; ^6^ Division of Allergy, Immunology and Transplantation, National Institute of Allergy and Infectious Diseases, Rockville, MD, United States

**Keywords:** asthma, children, epidemiology, memory immune response, infants, peripheral blood mononuclear cells, RSV (respiratory syncytial virus)

## Abstract

**Background:**

It is unknown whether RSV infection in infancy alters subsequent RSV immune responses.

**Methods:**

In a nested cohort of healthy, term children, peripheral blood mononuclear cells (PBMCs) were collected at ages 2-3 years to examine RSV memory T cell responses among children previously RSV infected during infancy (first year of life) compared to those RSV-uninfected during infancy. The presence *vs*. absence of infant RSV infection was determined through a combination of RSV molecular and serologic testing. Memory responses were measured in RSV stimulated PBMCs.

**Results:**

Compared to children not infected with RSV during the first year of life, children infected with RSV during infancy had lower memory T cell responses at ages 2-3 years to *in vitro* stimulation with RSV for most tested type-1 and type-17 markers for a number of memory T cell subsets.

**Conclusions:**

RSV infection in infancy has long-term effects on memory T cell responses. This is the first study to show the potential for RSV infection in infancy to have long-term effects on the immune memory irrespective of the severity of the infection. Our results suggest a possible mechanism through which infant RSV infection may result in greater risk of subsequent childhood respiratory viral morbidity, findings also relevant to vaccine development.

## Introduction

Respiratory syncytial virus (RSV) is a ubiquitous, seasonal pathogen that infects the ciliated epithelium of the upper and lower respiratory tracts and is the single most important cause of infant lower respiratory tract infections worldwide (1–5). Most children are infected by RSV by age 2 years, but RSV then causes repeat infections throughout life; the serious complications of infection are most common in infancy and in the elderly (4, 6, 7). Infant infection is likely to have the greatest impact on immune development, given that the infant’s immune system is rapidly developing. Thus, understanding the long-term effects of RSV infection during the critical period of the developing infant immune system is important in understanding and preventing RSV’s contribution to chronic respiratory morbidity (8–16). Many clinical studies of RSV’s role in long-term respiratory morbidity have focused on severe RSV infection, i.e. children hospitalized with RSV-associated bronchiolitis, compared to children without severe RSV disease but who may have been infected with RSV. This design may fail to capture initial life exposure to RSV and the full clinical spectrum of disease, thereby limiting systematic analyses of long-term effects of RSV infection on immune memory. For the current study, we used a nested cohort of a larger population-based cohort (17) where children were prospectively followed from early infancy throughout the first year of life, with RSV infection status determined by PCR of respiratory secretions during RSV season and RSV antibody studies at 1 year of age. The nested cohort allowed us to compare RSV memory T cells from 2-3 year old children who were infected with RSV during their first year of life to RSV memory T cells from children who remained uninfected at 1 year of age, enabling determination of whether RSV infection during infancy induces a different memory T cell response than infection later in life.

## Materials and Methods

### Overview of the Study Population and Design

**Figure 1:**
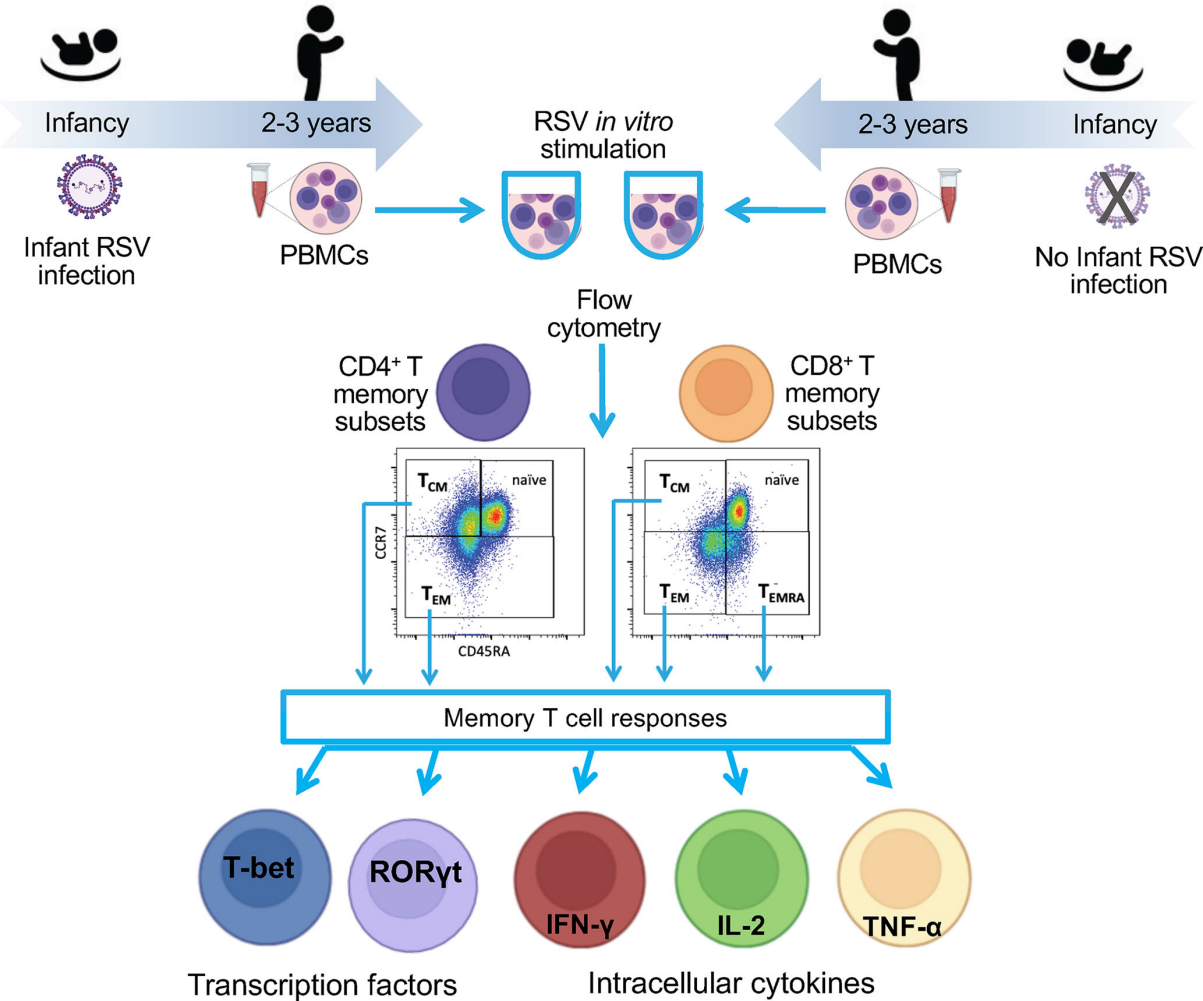
Flow diagram of the study. To understand the impact of RSV infection in infancy on RSV memory T cell responses, we collected blood from a nested cohort of 75 children enrolled in the INSPIRE cohort who were or were not infected with RSV during infancy. The PBMCs were collected at ages 2-3 years. PBMCs were stimulated *in vitro* with RSV, and functional responses were measured within memory CD4 and CD8 T cell subsets by percentage of cell expressing nuclear transcription factor or intracellular cytokines. Results were compared between children infected with RSV during infancy and those who were not.

The Infant Susceptibility to Pulmonary Infections and Asthma Following RSV Exposure study (INSPIRE) is a population-based, birth cohort of healthy, term children specifically designed to determine the presence *vs*. absence of RSV infection in infancy (i.e., the first year of life) on subsequent childhood respiratory morbidity. Eligible children were overall healthy, born at term and non-low birthweight, and enrolled near birth, between June and December of 2012 and 2013 (i.e. would be ≤6 months of age at the beginning of their first RSV season, November to March in our region) (10, 18). An *a priori* designed nested cohort of children with and without infant RSV infection included longitudinal measures of memory T cell responses at ages 2-3 years (completed) and age 6-7 years (ongoing) to understand the long-term impact of infant RSV infection on immune outcomes. The detailed methods for INSPIRE have been previously reported, and an overview of this study design is depicted in **Figure 1** (17). The Institutional Review Board of Vanderbilt University approved this study (IRB# 111299) and one parent of each child provided informed consent for their participation.

### Determination of RSV Infection in Infancy

For the ascertainment of RSV infection in infancy, we first conducted passive and active surveillance during each child’s first RSV season. In children who met pre-specified criteria for an acute respiratory illness, a nasal wash was tested for RSV using reverse transcription-quantitative PCR (17, 19). In addition, RSV serum antibody titers were measured in blood samples obtained at age 1-year by enzyme-linked immunosorbent assay (ELISA) as previously described (20). Briefly, the ELISA detects IgG antibodies to RSV in infected tissue culture lysates containing group A and B antigens; uninfected tissue culture lysate serves as a control for nonspecific antibody binding. Children were then grouped into those infected *vs*. not infected with RSV in infancy using a hierarchical categorization with mutually exclusive group membership.

### Nested Cohort for Peripheral Blood Mononuclear Cells (PBMCs) Studies

To understand the impact of RSV infection in infancy on RSV memory T cell responses, we collected blood from a nested cohort of 75 children enrolled in the INSPIRE cohort who were or were not infected with RSV during infancy. The PBMCs were collected at ages 2-3 years (median age [interquartile age] = 2.83 [2.25-3.83] years).

### PBMC Isolation

For PBMC isolation, up to ~30 ml of blood was collected using the BD Vacutainer^®^ CPT™ system (Becton Dickinson, New Jersey, United States) and PBMCs were separated according to the manufacturer’s instructions. PBMCs were subsequently counted, divided into aliquots, and stored in liquid nitrogen until further analyses.

### RSV Propagation and Purification

RSV A2001/3-12 (RSV 3-12) was propagated in HEp-2 cells and purified by ultracentrifugation through a 20% sucrose cushion as previously described (21, 22). Briefly, HEp-2 cells were maintained in MEM media (Gibco, Thermo Fisher Scientific, Massachusetts, USA) with 5% FBS (HyClone, Thermo Fisher Scientific, Massachusetts, USA), and cells were infected with a low passage stock RSV 3-12 virus at multiplicity of infection (MOI) = 0.01 TCID_50_/ml. The high titer virus was collected by snap-freezing and thawing the infected cultures, and supernatant was clarified by centrifugation at 1000 x g at 4C for 10 min. The clarified supernatant was overlayed on a 20% of sterile sucrose solution, and virus was pelleted by ultracentrifugation at 13,000 x g at 4C for 2 hours. The purified virus was re-suspended in serum-free MEM medium and stored at -80°C. The infectivity titer of purified RSV was assessed in a TCID_50_ assay in Hep-2 cells; the stimulation dose for PBMC experiments was an MOI of 1 TCID_50_.). Mock challenge material was prepared and purified in the same manner as RSV but with uninfected HEp-2 cultures.

### Memory T Cell Responses Assay

Freshly thawed PBMCs were washed and resuspended in RPMI medium supplemented with 2% human pooled AB serum (Sigma-Aldrich, Missouri, United States) and left for 6 hours at 37°C to rest. Following the initial rest, PBMCs were again washed and resuspended in fresh RPMI media supplemented with 2% human serum plus 20 ng/ml recombinant human IL-2 (R&D Systems, Inc., Minnesota, United States) and purified RSV 3-12 at MOI = 1 or similarly diluted mock. Staphylococcal enterotoxin B subunit (SEB) at the final concentration of 1 μg/ml was used as a positive control. PBMCs were incubated with RSV 3-12, mock, or SEB for 72 hours at 37°C, 5% CO2, with Brefeldin A (BD Biosciences, California, United States) added for the last 5 h of incubation, then washed, and stained for flow cytometry with Zombie Violet (Biolegend, California, United States), anti-CD3 (Biolegend), anti-CD4 (Biolegend), anti-CD8 (BD Biosciences), anti-CD45RA (eBioscience, Inc., California, United States), and anti-CCR7 fluorescent-labeled antibodies (BD Biosciences). Cells were fixed and permeabilized with transcription factor fixation/permeabilization solution (eBioscience) and permeabilization buffer (eBioscience) and stained intracellular with fluorescent-labeled antibodies against T-bet (Biolegend), GATA3 (eBioscience), RORγt (Biolegend), TNF-α (Biolegend), IFN-γ (Biolegend), and IL-2 (eBioscience). Flow cytometry data were obtained using a 4-laser BD LSR-II™ system (BD Biosciences) and analyzed with BD FACSDiva (BD Biosciences) and FlowJo software (Tree Star Inc, Oregon, United States). Memory T cell subsets were identified within live CD3+ CD4+ and CD3+CD8+ populations as central memory T cells (TCM, CD45RA^low^CCR7^hi^), effector memory T cells (TEM, CD45RA^low^CCR7^low^), and as effector memory T cells expressing CD45RA (TEMRA, CD45RA^hi^CCR7^low^). To determine functional phenotypes, within each memory T cell subpopulation we identified the percentage of cells expressing selected type-1 (IFN-γ, TNF-α, IL-2, and T-bet), type-2 (GATA3), and type-17 (RORγt) markers. Positive control SEB was used to gate positive marker expressing cells (**Figures S1**, **S2**). The mean of mock-stimulated wells was subtracted from the corresponding mean of RSV-stimulated replicates prior to statistical analyses.

### Statistical Analyses

We used unadjusted and adjusted generalized least squares regression models to determine the relationship of infants’ RSV infection status with the memory T cell responses while accounting for clustered data. The adjusted models controlled for the child’s sex and race or ethnicity. Statistical significance was defined as p<0.05. We report both negative and as well as positive results for the pre-specified the immune marker comparisons between RSV infected *vs* RSV uninfected status, and no adjustments were made for multiple tests.

Statistical analyses were performed using R version 3.6.1 (23). Figures were created using the R ggplot2 package (24), and GraphPad Prism version 8.4.3 (available at: https://www.graphpad.com/).

## Results

Our memory T cell studies were performed using PBMC collected from 75 children at ages 2-3 years enrolled in the nested cohort within the larger INSPIRE birth cohort. The demographics for this nested cohort are shown in **Table 1**. Children included in the INSPIRE cohort were followed after birth with their RSV status tested by PCR and serology throughout the first year of life. The detailed description of INSPIRE and RSV testing has been previously reported (17).

**Table 1:** Demographic characteristics of children in nested cohort.

	Nested cohort (all)	Infant RSV infection	No infant RSV infection
	(N = 75)	(N = 56)	(N = 19)
Sex			
Male	57%	57%	58%
Female	43%	43%	42%
Gestational age (median, IQR)	39.0 (38.0, 40.0)	39.0 (38.0, 39.48)	39.3 (39.0, 40.0)
Birth weight (median, IQR)	3462.00	3447.50	3490.00
	(3135.50, 3703.00)	(3135.75, 3689.00)	(3163.50, 3717.00)
Race and ethnicity			
White	55%	52%	63%
Black	27%	34%	5%
Hispanic	7%	5%	11%
Other	12%	9%	21%
Insurance, %			
Medicaid	60%	66%	42%
Private	39%	34%	53%
Other/unknown	1%	0	5%
Breast feeding (ever), %	79%	79%	79%
Daycare during infancy, %	41%	43%	37%
Siblings, %	43%	48%	26%

Fifty-six (75%) of the 75 study children (**Table 2**) had documented RSV infection during their first year of life, i.e. had RSV infection confirmed by either PCR or serology assay. Control subjects (25% of cohort) did not have documented RSV infection during their first year of life, although were likely subsequently infected during their second and/or third year and before the PBMCs were collected for this study. Hence our study focused on the effect of infant RSV infection on the T cell responses at 2-3 years of age, irrespective of the occurrence and frequency of the intervening RSV infections.

**Table 2:** Infant RSV infection status of children in nested cohort.

	Nested cohort (all)	Infant RSV infection	No infant RSV infection
	(N = 75)	(N = 56)	(N = 19)
Median RSV IgG antibody titer (IQR)	3743.5	5764	<200*
	(154.5, 7309.5)	(2571, 10736)	
RSV infection status			
RSV PCR +	73%	98%	0
RSV serology +	75%	100%	0
Both	73%	98%	0
Neither	25%	0	100%

*The assay limit detection is 200. Values <200 indicate no RSV antibody was detected.

The main memory T cell subsets, CD4 and CD8 TCM, TEM and TEMRA, and their functional cytokine responses were studied. PBMC were stimulated *in vitro* with the purified RSV virus and percentage of cells producing IFN-γ, IL-2, TNF-α, GATA3 and RORγt were determined within each memory T cell subset. Responses of children exposed to RSV in infancy (n=56) were compared to those who did not have documented RSV infection in the first year of life (n=19).

**Figure 2:**
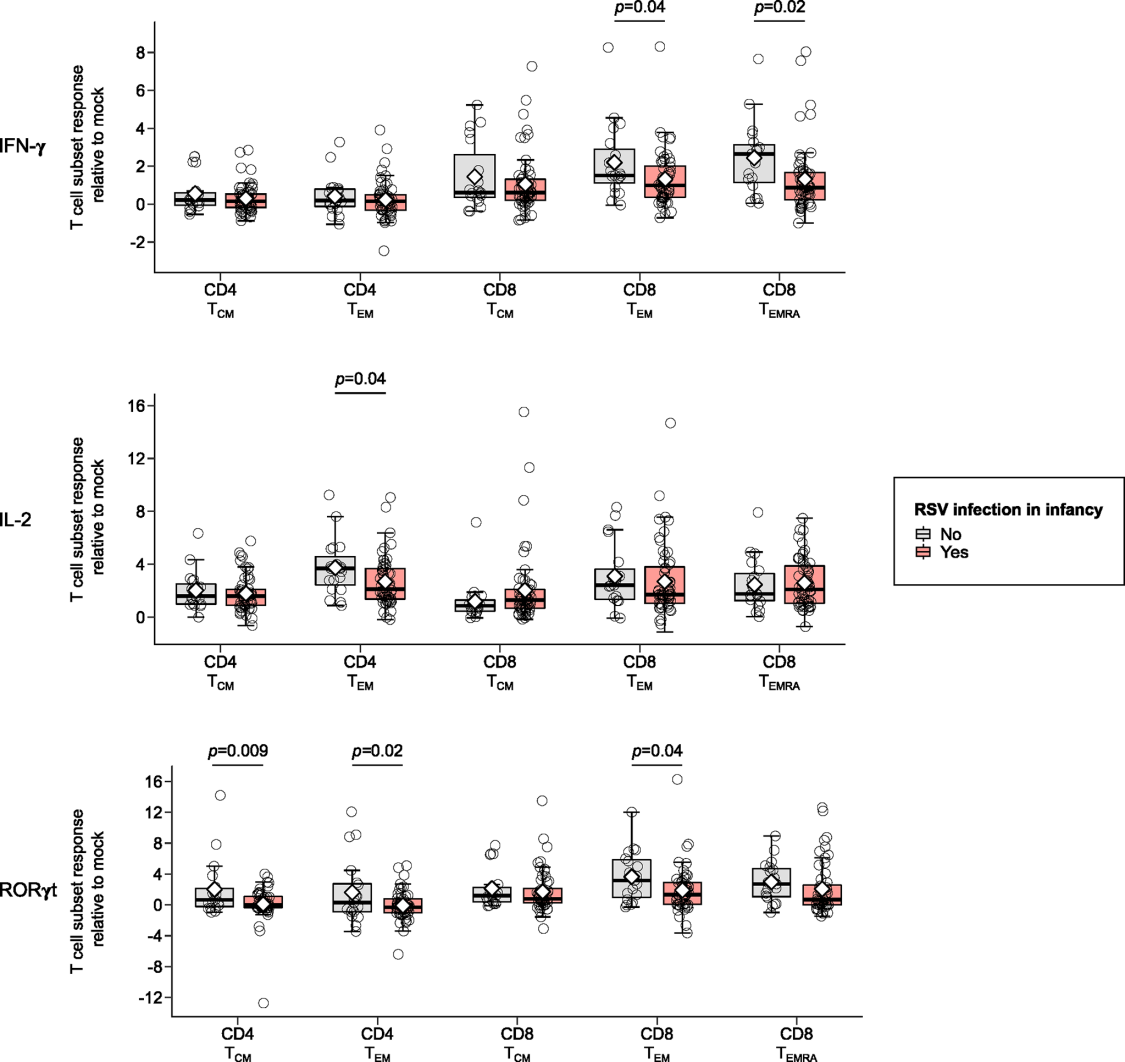
Memory T cell responses at ages 2-3 years following *in vitro* stimulation with RSV 3-12 according to RSV infection in infancy: IFN-γ, IL-2, and RORγt. The x-axis indicates the CD4 (TCM and TEM) and CD8 (TCM, TEM, and TEMRA) memory T cell subsets. The y-axis denotes the T cell subset response relative to mock for the indicated cytokines following *in vitro* stimulation with RSV for the frequencies of cells expressing IFN-γ, IL-2 and ROR-γt respectively. The box-and-whisker plots show the mean (diamond), median (middle bar), 1^st^ quartile (lower bar), 3^rd^ quartile (upper bar), minimum observation above the lower fence (lower whisker), maximum observation below the upper fence (upper whisker), and individual observations (circles) for each group. The significant p-values (<0.05) for the comparison between groups using generalized least squares regression to account for clustered data are also shown. Only the markers significantly differing between study groups for ≥1 memory T cell subsets are shown.

**Figure 3:**
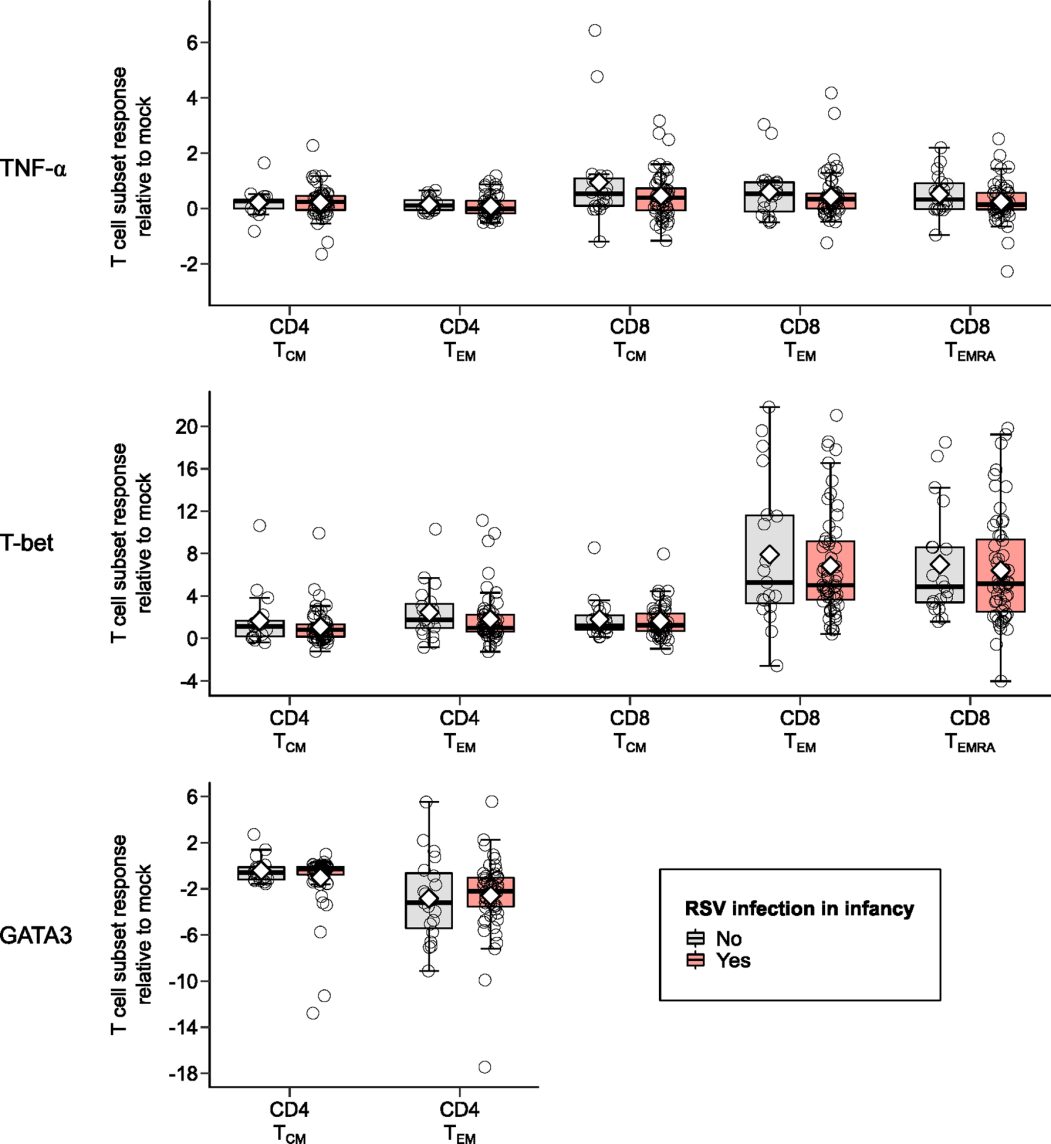
Memory T cell responses at ages 2-3 years after *in vitro* stimulation with RSV 3-12 according to RSV infection in infancy: TNF-α, T-bet, and GATA3. The x-axis indicates the CD4 (TCM and TEM) and CD8 (TCM, TEM, and TEMRA) memory T cell subsets. The y-axis denotes the T cell subset response relative to mock following *in vitro* stimulation with RSV. The box-and-whisker plots show the mean (diamond), median (middle bar), 1^st^ quartile (lower bar), 3^rd^ quartile (upper bar), minimum observation above the lower fence (lower whisker), maximum observation below the upper fence (upper whisker), and individual observations (circles) for each group. The significant p-values (<0.05) for the comparison between groups using generalized least squares regression to account for clustered data are also shown.

Children infected with RSV in infancy had significantly lower memory T cell responses to *in vitro* stimulation with RSV 3-12 for most tested type-1 (IFN-γ, IL-2) and type-17 (RORγt) markers (**Figure 2**). These associations remained significant in both unadjusted and adjusted models for a number of marker-expressing memory subsets, including CD8 TEM and CD8 TEMRA IFN-γ; CD4 TEM IL-2; and CD4 TCM, CD4 TEM, and CD8 TEM RORγt (**Table 3**). T-bet and TNF-α responses displayed decreasing trends in the effect sizes among children infected with RSV in infancy. Interestingly, the frequencies of GATA3-expressing cells trended slightly towards an increase in the infant RSV-positive group but the effect size was relatively small. Differences in T-bet, TNF-α and GATA3 were not found to be statistically significant (**Figure 3**).

**Table 3:** The association of the absence of RSV infection in infancy with memory T cell responses at ages 2-3 years after *in vitro* stimulation with RSV.^*†^.

Immune marker	T cell subset	*Unadjusted analyses*		*Adjusted analyses* ^‡^	
		β (95% CI)	*p*-value	β (95% CI)	*p*-value
IFN-γ	CD8^+^ T_EM_	0.88 (0.05 to 1.70)	0.04	0.86 (0.02 to 1.69)	0.04
	CD8^+^ T_EMRA_	1.12 (0.19 to 2.05)	0.02	1.03 (0.11 to 1.96)	0.03
IL-2	CD4^+^ T_EM_	1.06 (0.06 to 2.06)	0.04	1.05 (0.03 to 2.06)	0.04
RORγt	CD4^+^ T_CM_	1.89 (0.47 to 3.31)	0.009	1.83 (0.40 to 3.27)	0.01
	CD4^+^ T_EM_	1.70 (0.27 to 3.12)	0.02	1.66 (0.22 to 3.11)	0.02
	CD8^+^ T_EM_	1.75 (0.14 to 3.36)	0.03	1.68 (0.05 to 3.31)	0.04

CI, Confidence interval; RSV, Respiratory syncytial virus; T_CM_, T central memory cells; T_EM_, T effector memory cells; T_EMRA_, T effector memory cells expressing CD45RA.

^*^The estimates were obtained from unadjusted and adjusted generalized least squares regression models to account for clustered data. The reference group was children with RSV infection in infancy. The β estimate denotes the effect of the absence of RSV infection in infancy on a T cell subset response relative to mock following the in vitro stimulation with RSV for a priori selected type-1 (IFN-γ and IL-2) and type-17 (RORγt) immune markers. Only associations that were significant in bivariate analyses are shown.

^†^The statistical analyses were conducted in children with complete data.

^‡^The adjusted models included child’s sex and race or ethnicity as covariates.

## Discussion

This is the first study to show an effect of infant RSV infection on the RSV memory T cell response later in life, irrespective of the severity of the first RSV infection in infancy, and of the occurrence and frequency of any subsequent RSV infections. The population-based INSPIRE birth cohort (almost 2000 children enrolled at birth) that included longitudinal PCR surveillance for RSV during the first year of life, and RSV serology at 1 year to capture any missed infections, allowed us to achieve a fairly large number of participants with documented presence or absence of RSV infection during infancy to conduct T cell response assessment in subsets with and without infant RSV infection. Blood collection at 2-3 years of age enabled us to comprehensively evaluate T cell responses imprinted by the infant (first year of life) RSV exposure and persisting later in life irrespective of subsequent infections, leading to the first evidence from human studies that RSV memory T cell responses differ depending on when during early life infection occurred.

Our data show that infant RSV infection dampens T cell responses regardless of the severity of infection or subsequent re-infections with RSV. To our knowledge, only one other similar study has been conducted (25). In contrast to ours, this study found that PBMCs from children with RSV infection in infancy produce higher levels of IFN-γ and IL-2 following *in vitro* RSV infection later in life. However, this study was small (n=18), only assessed undifferentiated PBMC responses just beyond infancy at age ~1.2 years, and did not include statistical comparisons (25). The role of RSV infection in infancy on later childhood respiratory morbidity is also supported by numerous *in vitro* and animal studies that provide evidence for potential mechanisms through which early-life RSV infection may contribute to chronic airway diseases by impairing regulatory T cell function (15, 26, 27). A study by Tusker et al. using a murine model of RSV infection demonstrated differences in long-term T cell responses between animals infected with RSV as neonates and those exposed to the primary RSV infection as adults (28). Similar to our clinical findings, neonatal mice infected with RSV exhibited impaired type-1 responses to secondary RSV infection in adulthood. Interestingly, even though neonatally infected mice developed protection against later re-infection with RSV, they had significantly lower expression of IFN-γ and higher levels of IL-4 and IL-5 (type-2 responses) compared to animals primed as adults. Our findings suggest that RSV infection in infancy attenuates later antiviral type-1 and type-17 memory responses. Although frequencies of GATA3-expressing cells rose slightly following RSV stimulation of PBMCs from children infected during infancy, we did not observe significant increases in this measure of Th2 responses among these participants. However, it is possible that RSV infection in infancy could alter expression of other markers of CD4 Th2 and/or affect CD8 Th2 responses.

Age-related differences in innate cytokine responses to RSV infection have been demonstrated, and the importance of this early response is supported by polymorphisms in many early innate genes associated with severe RSV infection (29). Genetic predisposition may result in the dampened Th1 responses to RSV and skewing toward the Th2 and Th17 polarization contributing to other respiratory morbidity, although not all studies demonstrate that infant RSV infection results in a Th2-biased memory. We observed significantly higher frequencies of RORγt-expressing T cells obtained from children who did not have RSV infection in infancy as compared to early-life RSV-infected participants. Some clinical data suggest a definitive role of type-17 responses in the less severe outcomes or improved recovery from RSV infection (30). Infants 6 months and younger with moderate infection were shown to have higher IL-17 plasma levels then infants with severe RSV disease (31), and IL-17 levels were significantly elevated in non-ventilated *vs*. ventilated RSV-infected pediatric patients at the point of hospital discharge (32). Several animal studies also demonstrate significant differences in antiviral memory T cell responses based on age of primary RSV infection, which together with our results, highlight early life as a critical window impacting memory T cell responses (28, 33).

We note some limitations of our study. First, we cannot completely exclude misclassification of children categorized as not infected with RSV in infancy. However, this likely would have biased our results towards the null. Due to the cost and labor-intensive nature of these studies the sample size was small, thus precluding adjusting for multiple covariates. Other viral infections may also play a role, even though we assessed cell responses strictly after RSV stimulation, we cannot exclude the possibility that other pathogens or vaccines might have affected the responses we measured. It is possible that the higher PBMC responses we detected in children not infected during the first year resulted from the memory inducing infection being more recent. Though a possible contributor, we do not feel this would have had a substantial impact on results. All children would have been RSV exposed, both previously infected and RSV naïve children would have been infected, or re-infected, and, thus, reinfections might have boosted memory responses in some of the children infected during their first year of life. Note that that majority (76%) of the study PBMCs were collected between April and October outside the RSV season and would not have acutely responding T cells. We also excluded acute RSV infection at the time of collection. Finally, there was a mix of response differences including no differences, increases and decreases in response levels between the two groups. Though our sample size is limited, it is, nevertheless, relatively large when considering other studies of childhood T cell immune responses to RSV (34–39). Lastly, our study does not address the basis of age associated patterns of T cell responses to early-life RSV infection, though we recognize that immune responses to viral infection do not occur in isolation and that other host-pathogen interactions such as the airway epithelial cell responses and the microbiome may contribute to differences in our clinical outcomes.

In summary, children who were infected with RSV during their first year of life have dampened antiviral memory T cell responses to RSV at age 2-3 years compared to children who were not infected with RSV during their first year of life, suggesting that early life infection shapes the developing immune system. These results highlight the impact of infant RSV infection may have on anti-viral immunity. It is possible that alterations in the RSV memory response affect responses to other viral infections and explain in part the enhanced susceptibility to respiratory viral infection and virus-triggered asthma exacerbations. Also, the influence of RSV infection on the developing immune system, and memory T cells responses in particular, may be relevant to other immune responses including vaccine associated responses.

## Data Availability Statement

The raw data supporting the conclusions of this article will be made available by the authors, without undue reservation.

## Ethics Statement

The Institutional Review Board of Vanderbilt University approved this study (IRB# 111299) and one parent of each child provided informed consent for their participation. Written informed consent to participate in this study was provided by the participants’ legal guardian/next of kin.

## Author Contributions

TC, CR-S, TG, WD, RP, TH, and LA contributed to the conception of the study and/or the study design. RP, LA, and TH obtained the research funding supporting this study. TC, SJ, JC, DN, and LA contributed to the sample processing and laboratory assays. TC, CR-S, TG, WD, and TH conducted the statistical analyses. All authors contributed to the interpretation of the data. TC, CR-S, TG, WD, TH, and LA wrote the initial version of the manuscript. All authors reviewed this initial version, revised it critically for important intellectual content, and approved the final version. All authors agree to be accountable for all aspects of the work in ensuring that questions related to the accuracy or integrity of any part of the work are appropriately investigated and resolved. All authors contributed to the article and approved the submitted version.

## Funding

This work was supported in whole or in part with funds from the National Institute of Allergy and Infectious Diseases (under award numbers U19AI095227 and K24AI77930); the Vanderbilt Institute for Clinical and Translational Research (grant support from the National Center for Advancing Translational Sciences under award number UL1TR000445); the National Heart, Lung, and Blood Institute (under award number K23HL148638); the Parker B. Francis Fellowship Program; and the Department of Pediatrics at Vanderbilt University Medical Center (grant support from the Eunice Kennedy Shriver National Institute of Child Health and Human Development under award number K12HD087023).

## Author Disclaimer

The content is solely the responsibility of the authors and does not necessarily represent the official views of the funding agencies.

## Conflict of Interest

LA has served on respiratory syncytial virus (RSV) vaccine advisory boards for Bavarian Nordic, Novavax, Daiichi-Sankyo, ClearPath Vaccines Company, ADVI, and Pfizer. Through Emory University, his laboratory currently receives funding from Pfizer for surveillance studies of RSV infection in adults, from Advaccine Biopharmacueticals Suzhou Co., Ltd. For serologic studies of RSV vaccine recipients, and from Sciogen for animal studies on RSV vaccines. He is a co-inventor on several Centers for Disease Control and Prevention patents on the RSV G protein and its CX3C chemokine motif relative to immune therapy and vaccine development. He is also co-inventor on a patent filing for the use of RSV platform virus-like particles with the F and G proteins for vaccines. TH has served on RSV vaccine advisory boards for Sanofi-Pasteur and Pfizer.

The remaining authors declare that the research was conducted in the absence of any commercial or financial relationships that could be construed as a potential conflict of interest.

## Publisher’s Note

All claims expressed in this article are solely those of the authors and do not necessarily represent those of their affiliated organizations, or those of the publisher, the editors and the reviewers. Any product that may be evaluated in this article, or claim that may be made by its manufacturer, is not guaranteed or endorsed by the publisher.

## Supplementary Material

Supplementary Figure 1 Flow cytometry gating strategy for PBMC studies. PBMCs collected at age 2-3 years were stimulated with clinical strain RSV A2001/3-12 at MOI = 1 for 72 h. Cells were washed, stained for surface markers, fixed, permeabilized and stained intracellular with fluorescent-labeled antibodies to detect intracellular IFN-γ, TNF-α, IL-2, T-bet, and RORγt. Percentage of cells expressing these cytokines and transcription factors were determined within CD4 and CD8 memory T cell subsets: TCM, TEM, and TEMRA. CD4 and CD8 memory T cell subsets were gated based on CCR7 and CD45RA expression within CD3 positive cells after consecutive doublet, nonlymphocyte and dead cell exclusion. Gating for cells expressing cytokines or transcription factors was performed using SEB-stimulated same specimen replicates as a positive gate control and gated naive T cell population of the same-specimen replicates as a negative gate control.

Supplementary Figure 2 Representative flow cytometry plots of cells expressing cytokine and transcription factors in PBMCs after *in vitro* stimulation with RSV 3-12. PBMCs collected at age 2-3 years were stimulated with clinical strain RSV A2001/3-12 at MOI =1 for 72 h. Cells were washed, stained for surface markers, fixed, permeabilized and stained intracellular with fluorescent-labeled antibodies to detect intracellular IFN-γ, TNF-α, IL-2, T-bet, and RORγt. Percentages of cells expressing these cytokines and transcription factors were determined within CD4 and CD8 memory T cell subsets by flow cytometry (plots show CD4 and CD8 TEM subsets).

## Abbreviations

CI, Confidence interval; INSPIRE, Infant Susceptibility to Pulmonary Infections and Asthma Following Respiratory; Syncytial Virus Exposure Study; PBMCs, Peripheral blood mononuclear cells; RSV, Respiratory syncytial virus; TCM, T central memory cells; TEM, T effector memory cells; TEMRA, T effector memory cells expressing CD45RA; IFN-γ, Interferon gamma; TNF-α, Tumor necrosis factor alpha; IL-2, Interleukin 2; T-bet, T-box expressed in T cells; GATA3, G-A-T-A 3 binding protein; RORγt, Retinoic acid-related orphan receptor gamma t.
