# Supplementary figures and images for: Effect of Infant RSV Infection on Memory T Cell Responses at Age 2-3 Years
Source: Front Immunol. 2022 Mar 17;13:826666. doi: 10.3389/fimmu.2022.826666 (PMC8967987; doi:10.3389/fimmu.2022.826666)

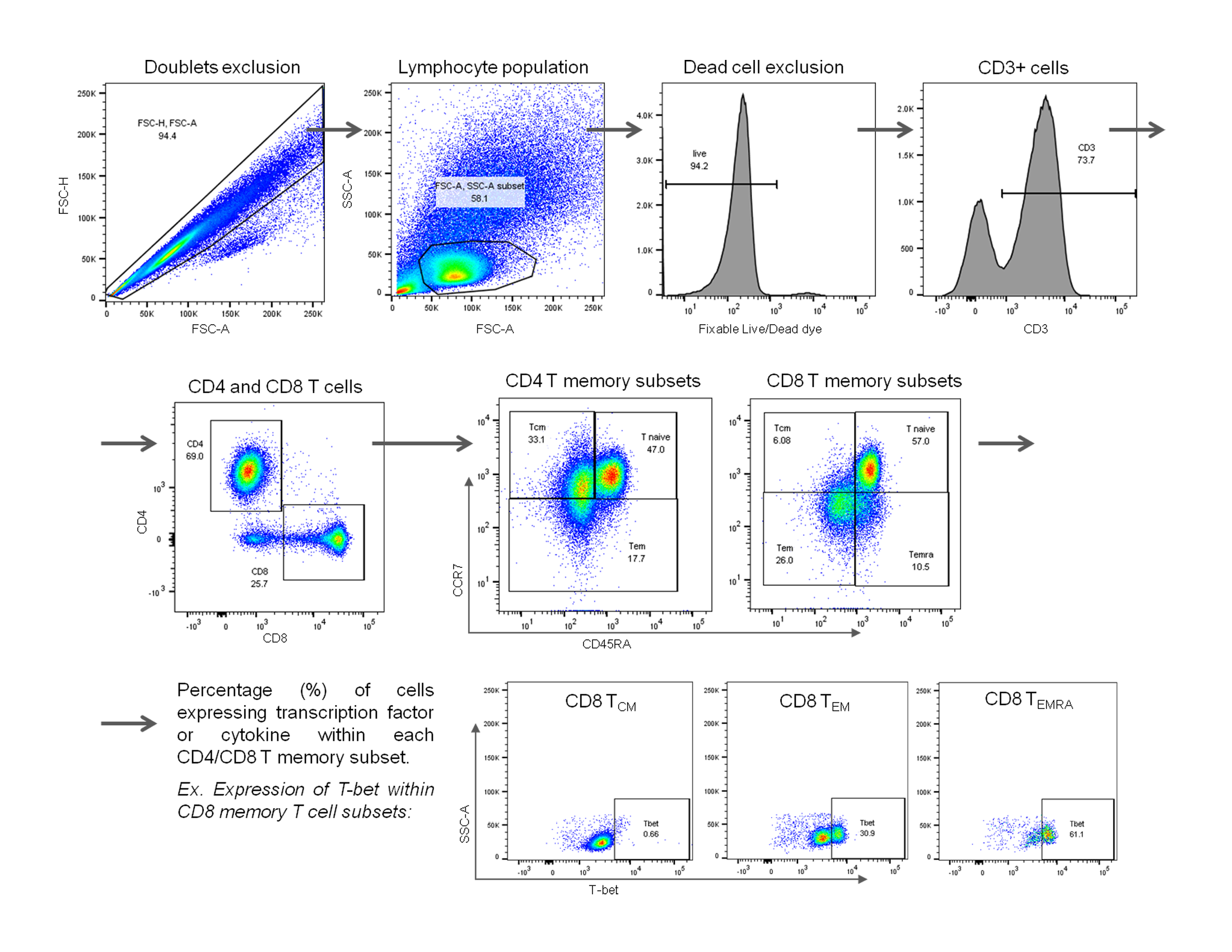

Supplement: Supplementary Figure 1 — Flow cytometry gating strategy for PBMC studies. PBMCs collected at age 2-3 years were stimulated with clinical strain RSV A2001/3-12 at MOI = 1 for 72 h. Cells were washed, stained for surface markers, fixed, permeabilized and stained intracellular with fluorescent-labeled antibodies to detect intracellular IFN-γ, TNF-α, IL-2, T-bet, and RORγt. Percentage of cells expressing these cytokines and transcription factors were determined within CD4 and CD8 memory T cell subsets: TCM, TEM, and TEMRA. CD4 and CD8 memory T cell subsets were gated based on CCR7 and CD45RA expression within CD3 positive cells after consecutive doublet, nonlymphocyte and dead cell exclusion. Gating for cells expressing cytokines or transcription factors was performed using SEB-stimulated same specimen replicates as a positive gate control and gated naive T cell population of the same-specimen replicates as a negative gate control. [file Image_1.tiff]

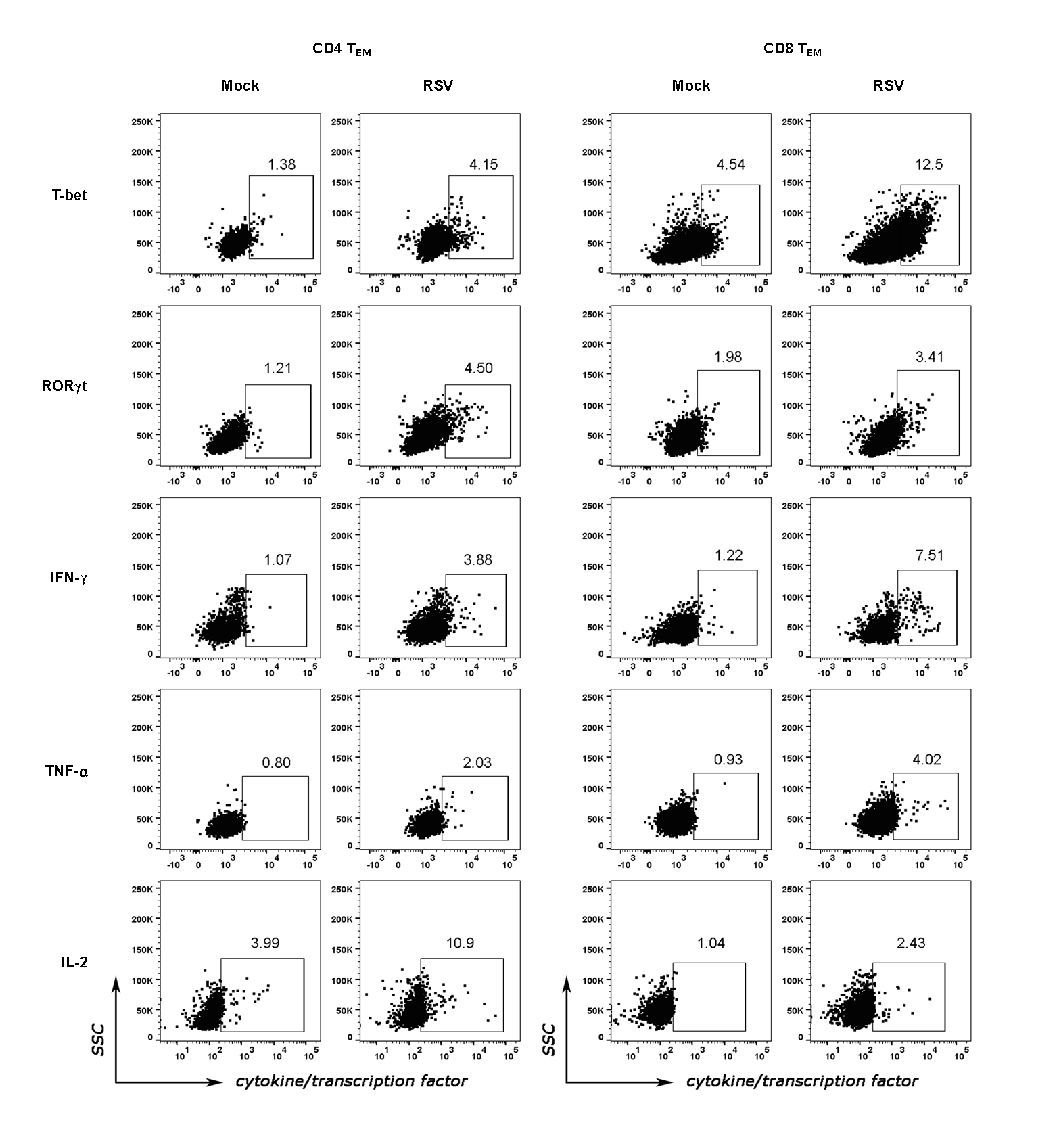

Supplement: Supplementary Figure 2 — Representative flow cytometry plots of cells expressing cytokine and transcription factors in PBMCs after in vitro stimulation with RSV 3-12. PBMCs collected at age 2-3 years were stimulated with clinical strain RSV A2001/3-12 at MOI =1 for 72 h. Cells were washed, stained for surface markers, fixed, permeabilized and stained intracellular with fluorescent-labeled antibodies to detect intracellular IFN-γ, TNF-α, IL-2, T-bet, and RORγt. Percentages of cells expressing these cytokines and transcription factors were determined within CD4 and CD8 memory T cell subsets by flow cytometry (plots show CD4 and CD8 TEM subsets). [file Image_2.tiff]
